# HOW GRANDPARENT COUPLES ENGAGE IN FAMILY LEISURE WITH GRANDCHILDREN

**DOI:** 10.1093/geroni/igad104.0857

**Published:** 2023-12-21

**Authors:** Jill Juris, Anisa Zvonkovic

**Affiliations:** Appalachian State University, Boone, North Carolina, United States; University of Georgia, Athens, Georgia, United States

## Abstract

There is limited understanding of how grandparent couples negotiate time with grandchildren. Family leisure is often purposive by being planned, facilitated, and executed by parents or grandparents to achieve goals such as improved interaction, communication, and family cohesion. Moving beyond studies of counting time in leisure, few studies of family leisure have included how people experience leisure as couples. Guided by a life course approach, this qualitative study provides insight to an understanding of the process of family leisure among grandparent couples by examining how both individuals in a couple experience family leisure with their grandchildren. Grandparent couples (n=10) ranged in age from 60 to 75 years old (M=66.8, SD=5.01). The grandparents interviewed were not providing custodial care to their grandchildren. Participants completed individual interviews that were then analyzed at the couple level. Through constructivist grounded theory, this study developed a process model of grandparents’ decision-making that unfolded into four options for engaging in family leisure with grandchildren. The four options included: wanting a grandparent fix, going with the flow when invited by children, hosting grandchildren in their homes, and meeting expectations for family gatherings. Grandparent couples often described more than one option of family leisure based on their life course dimensions (i.e., individual, family, and sociohistorical). Implications for application in the fields of recreation and family sciences will be discussed.

